# Cartridge-based nucleic amplification (CBNAAT)/GeneXpert test as a diagnostic modality for the detection of genital tuberculosis in women with infertility

**DOI:** 10.3205/id000093

**Published:** 2025-07-11

**Authors:** Chavini K. Shaozae, Yogita Rai, Nishtha Jaiswal, Manoj B. Jais

**Affiliations:** 1 Department of Microbiology, Lady Hardinge Medical College and Associated SSK and KSC Hospitals, New Delhi, India; 2 Department of Obstetrics and Gynaecology, Lady Hardinge Medical College and Associated SSK and KSC Hospitals, New Delhi, India

**Keywords:** culture, genital tuberculosis, GeneXpert, infertility, Mycobacterium tuberculosis

## Abstract

**Background:**

Genital tuberculosis (GTB) is a significant etiological factor of infertility in developing countries such as India; however, it is frequently undiagnosed due to its asymptomatic nature and a lack of standardised protocols. This study aimed to compare the diagnostic efficacy of GeneXpert (CBNAAT) with Ziehl-Neelsen (ZN) staining, Mycobacterial Growth Indicator Tube (MGIT) liquid culture and histopathological examination (HPE). Additionally, the occurrence of GTB in infertile women aged between 18 and 45 years was also determined.

**Methods:**

The study comprised 200 infertile women with suspected GTB. Endometrial biopsy samples were collected aseptically and subjected to ZN staining, MGIT liquid culture, GeneXpert testing and HPE and the results were analysed and compared. MGIT was considered the gold standard test in accordance with National TB Elimination Programme (NTEP) recommendations.

**Results:**

There were 164 (82%) cases of primary infertility, and 36 (18%) cases of secondary infertility. Out of the 200 samples of endometrial biopsy (EB) specimens, the GeneXpert test detected two positive findings (1%), ZN staining detected two positive results (1%), and MGIT liquid culture as well as HPE detected one positive result (0.5%). GeneXpert demonstrated a sensitivity of 100% (confidence interval (CI) 2.50–100.00%), a specificity of 99.5% (CI 97.23–99.99%), a positive predictive value (PPV) of 50% (CI 12.40–87.60%), and a negative predictive value (NPV) of 100% (CI 98.15–100.00%), with liquid culture as reference. A significant agreement was found between the diagnostic procedures of MGIT and GeneXpert, with a kappa value of 0.66 and a p-value of 0.047 (significant p-value <0.05).

**Conclusion:**

The present study is among the few that has utilised GeneXpert to aid in the diagnosis of female genital tuberculosis (FGTB). GeneXpert, being much faster and more feasible than conventional methods such as culture, could be incorporated into the standard evaluation of GTB.

## Introduction

Tuberculosis (TB), a chronic infectious disease caused by *Mycobacterium tuberculosis* (MTB), is spread through the air from person to person. In the last few decades, it has been seen that the incidence of TB has increased due to the HIV pandemic, drug abuse, alcohol use disorder, tobacco smoking and poverty [1]. As per the Global Tuberculosis Report 2024, TB presumably regained its rank as the most fatal infectious agent-related cause of mortality worldwide in 2023, after three years during which it was surpassed by coronavirus disease (COVID-19) [2]. Although TB was discovered hundreds of years ago, it still remains one of the leading causes of death and a major health problem, especially in developing countries [3].

Extrapulmonary TB may involve all the organs and systems of the body, including the genital tract. Genital TB usually occurs secondary to primary infection, which most commonly involves the lungs. It generally spreads through lymphatic or hematogenous routes [4]. Genital tuberculosis (GTB) accounts for 9% of extra-pulmonary tuberculosis (EPTB) and continues to pose a challenge in the medical field due to the silent nature of the disease and lack of standard guidelines [5]. In a developing country like ours, GTB is an important etiological factor of infertility but often goes undiagnosed as it is commonly asymptomatic.

The World Health Organization (WHO) defines infertility as the failure to achieve pregnancy after 12 months or more of regular unprotected sexual intercourse [6]. The worldwide incidence of GTB has been found to be approximately 5–10% in infertile women. It ranges from 0.69% in some developed countries to even 19% in India [7].

Although the gold standard for a definitive diagnosis of EPTB is the isolation of MTB from samples by culture, in the past decade, there has been a shift of focus to newer, more rapid molecular technologies such as NAAT (Nucleic Acid Amplification Techniques) [8]. Culture methods are advantageous due to their higher sensitivity in detecting fewer bacilli (10–100 bacilli/ml of concentrated material), albeit time consuming as it can take up to 2–6 weeks [9]. 

In December 2020, the WHO recommended the use of GeneXpert MTB/RIF (Xpert; Cepheid, Sunnydale, CA), a real-time polymerase chain reaction (PCR) cartridge-based assay that can provide results within two hours, for the detection of TB and rifampicin resistance (RR) in areas with a high prevalence of multidrug-resistant tuberculosis (MDR TB) and human immunodeficiency virus and tuberculosis (HIV/TB) co-infection [8], [10].

In resource-constrained settings, the diagnosis of GTB is complicated by a number of factors, including the scarcity of laboratory facilities, the lack of sensitivity of routine, less expensive microbiological procedures such as ZN staining, the requirement for invasive procedures to obtain suitable samples, and the absence of standardised approaches for diagnosing the disease. The main objective of the study was to evaluate GeneXpert as a diagnostic tool in comparison with the conventional methods such as acid-fast bacilli (AFB) microscopy (ZN staining), Mycobacterial Growth Indicator Tube (MGIT) liquid culture, and histopathology to detect GTB in endometrial samples. This study also aimed to determine the occurrence of GTB in women with infertility aged between 18 and 45 years in a tertiary care centre.

## Methods

### Setting

This cross-sectional observational study was conducted between November 2022 and February 2024 for a period of 16 months in the Department of Microbiology in collaboration with the Department of Obstetrics and Gynaecology at a tertiary care hospital in New Delhi. The inclusion criteria included consenting females clinically diagnosed with primary and secondary infertility and those aged between 18 and 45 years. The exclusion criteria included patients with primary and secondary infertility, in whom the cause was confirmed to be other than female genital tuberculosis (FGTB), and those who were on Anti-Tubercular Therapy (ATT) for the last 6 months. Demographic data, clinical symptoms, and a detailed history were documented in a pre-approved proforma. 

### Diagnosis

**Figure 1:**
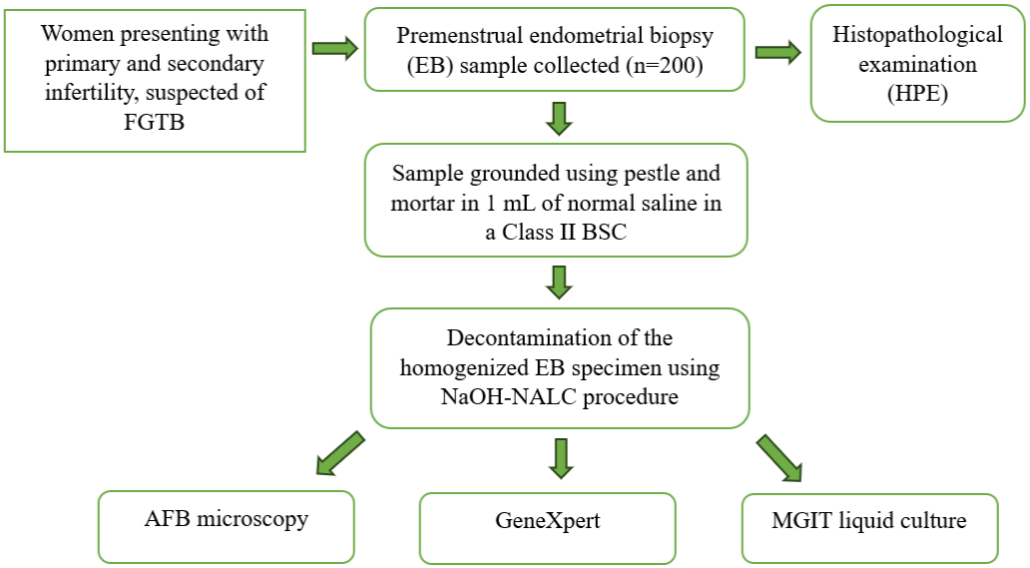
Flowchart depicting the diagnostic process of detecting AFB in endometrial biopsy (EB) specimens in patients suspected of FGTB

Premenstrual endometrial biopsy (EB) specimens of 200 infertile women were collected aseptically in sterile containers. Upon obtaining the samples in the laboratory, they were ground using a mortar and pestle in 1 ml of normal saline and decontaminated using the N-acetyl-l-cysteine–sodium hydroxide (NaOH-NALC) procedure [11]. The homogenised and decontaminated sediment was subjected to AFB microscopy, MGIT liquid culture, and GeneXpert testing. The endometrial biopsy (EB) specimens were also sent for histopathological examination (HPE). A simple flowchart illustrating the diagnostic process is shown in Figure 1 (Fig. 1).

### Sample size calculation

The sample size was estimated on the basis of reference studies that reported the prevalence of GTB in women presenting with primary and secondary infertility in North India to be 14% [8]. As a result, we chose P=4%, a confidence interval of 95%, and a margin error of 5%. The sample size was calculated using the following formula: n=([Z^2^×P×q]/e^2^), where n=sample size, Z=1.96 (95% confidence interval), P=estimated prevalence, q=1-P, and e=error [12]. Hence, the total calculated sample size was 186. For the study, a sample size of 200 was used.

### Statistical analysis

Data was entered in a Microsoft excel spreadsheet and analysed using EpiInfo 7.0. Continuous variables were depicted as mean (±SD) and median, while categorical variables were represented as frequency and percentage. An inter-rater reliability analysis was performed between MGIT and GeneXpert and/or ZN staining. For this purpose, the Cohen’s Kappa was calculated, which is a measure of the agreement between two dependent categorical samples. P value <0.05 was considered statistically significant. Cohen’s Kappa values are interpreted as follows: ≤0 indicates no agreement, 0.01–0.20 as none to slight, 0.21–0.40 as fair, 0.41–0.60 as moderate, 0.61–0.80 as significant, and 0.81–1.00 as almost perfect agreement.

## Results

### Demographics

**Figure 2:**
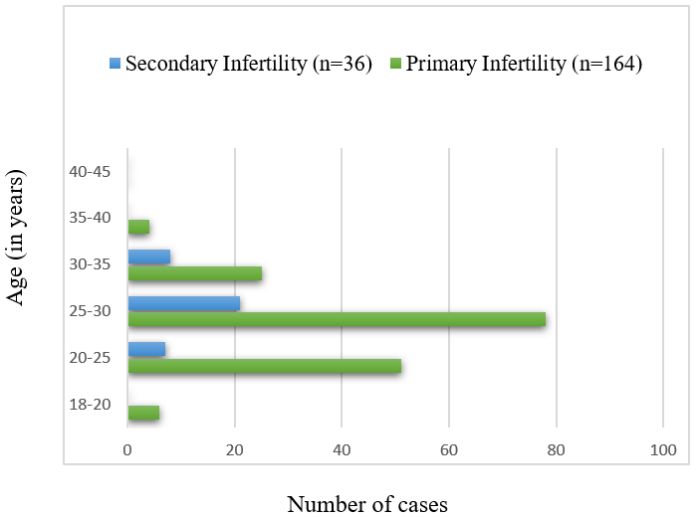
Distribution of primary and secondary infertility among patients with suspected FGTB (n=200) Legend: The X-axis represents the number of cases during the 16-month study period, while the Y-axis represents the age categories of the female patients (ranging from 18 to 45 years).

The mean age of the enrolled patients was 27.56 (±3.98) years and the mean duration of infertility was 5.11 (±3.33) years. The length of infertility ranged from 1 year to as long as 17 years. 82% (164/200) of the enrolled women with suspected FGTB had primary infertility, and 18% (36/200) had secondary infertility. Figure 2 (Fig. 2) depicts the distribution of primary and secondary infertility among the various age groups of the enrolled patients. 

### Diagnosis

Table 1 (Tab. 1) indicates the yield by GeneXpert, AFB microscopy, MGIT liquid culture, and histopathological findings, out of the 200 samples of EB specimens. Among the two positive outcomes, GeneXpert accurately detected one, while the other was a false positive, with MGIT and histopathology as reference. 

All individuals that tested positive for GeneXpert were found to be sensitive to rifampicin. Very low levels (as indicated by the GeneXpert software) of MTB were found in both positive cases using GeneXpert. The remaining findings were all correctly recognised as negative. Regarding ZN staining, both positive results were erroneous. Histopathological examination of the EB sample of one patient revealed Langhan’s giant cells, indicative of TB, which also came out to be positive by MGIT.

The vast majority of subjects (99.5%) tested negative for genital TB using the gold standard MGIT liquid culture method. Only 0.5% of the studied population tested positive by culture, as depicted in Tab. 4. The overall prevalence of genital TB confirmed by the gold standard method in the studied population was, therefore, 0.5% at our institution.

### Comparison of GeneXpert MTB/RIF, MGIT and AFB microscopy, used for the diagnosis of GTB

Table 2 (Tab. 2) shows the sensitivity, specificity, positive predictive value (PPV) and negative predictive value (NPV) of GeneXpert and microscopy, when performed on endometrial biopsy samples from individuals suspected of having FGTB, taking MGIT and histopathology as references. 

The Cohen’s Kappa was calculated between the variables GeneXpert and MGIT, with a significant p-value of 0.05. The Cohen’s Kappa showed that there was a substantial agreement between MGIT and GeneXpert with κ=0.66. A p-value of 0.047 indicated that the observed level of agreement (Kappa=0.66) is statistically significant (Table 3 (Tab. 3)). In other words, there is a low probability that the observed agreement is due to random chance. 

The Cohen’s Kappa was also calculated between the variables, AFB microscopy and MGIT, with a conventional threshold of p-value of 0.05. The Cohen’s Kappa showed that there was no agreement between MGIT and AFB microscopy with κ=–0.01. A p-value of 1 indicated that the observed level of agreement (Kappa=–0.01) is statistically insignificant (Table 4 (Tab. 4)). 

## Discussion

The World Health Organization (WHO) defines infertility as the failure to achieve pregnancy after 12 months or more of regular unprotected sexual intercourse. Infertility can either be primary or secondary. Primary infertility is when a pregnancy has never been achieved by a couple and secondary infertility is when there has been at least one conception before [7].

Our study included infertile women from 18 years of age until 45 years of age with a mean age of 27.56 (±3.98) years of the enrolled patients. The age group exhibiting the greatest proportion of individuals experiencing infertility was between 25 and 30 years old, constituting 49.5% of the overall population. Subsequently, it was followed by those in the age brackets of 20–25 years (29%), 30–35 years (16.5%), 18–20 years (3%), 35–40 years (2%), and none above 40 years of age, as illustrated in Figure 2 (Fig. 2). This finding is similar to that of Patel et al., who mentioned that infertility primarily impacted those under the age of 40, with the highest incidence occurring between the ages of 21 and 30 [13].

Among the 200 women who participated in our study, who were suspected of having FGTB and presented with infertility, 164 of them (82%) were diagnosed with primary infertility, while 36 of them (18%) were diagnosed with secondary infertility. The mean duration of infertility in our study was 5.11 (±3.33) years. This finding is comparable to that of Sethi et al., who conducted a similar investigation at Vardhman Mahavir Medical College and Safdarjung Hospital in New Delhi. In their study, 70% of the patients exhibited primary infertility, while 30% experienced secondary infertility, with a mean duration of infertility of 5.11 (±2.52) years [14].

Our study revealed that out of the 200 samples of EB specimens, the GeneXpert test detected two positive findings (1%), AFB microscopy (ZN staining) detected two positive results (1%), and MGIT liquid culture detected one positive result (0.5%). 

Furthermore, out of the two favourable results, GeneXpert successfully identified one, while the other turned out to be a false positive with MGIT as a reference as well as histopathology. All of the remaining findings were accurately identified as negative. On the other hand, both positive results from ZN staining were false positive. GeneXpert identifies DNA from nonviable cells, indicating that dead bacteria account for false positive results. This could be the reason for the false positive result in our study. According to Theron et al., GeneXpert may generate false-positive results for a period of up to four years following the completion of appropriate treatment [15]. False positives may lead to needless treatment with toxic medications, possibly delaying the actual diagnosis and its suitable therapy, and increasing healthcare expenses, as well. Therefore, clinicians must recognise the potential for false-positive GeneXpert results and evaluate the clinical context and carry out further diagnostic tests, such as repeated testing and culture, prior to commencing TB therapy [16]. As for false positivity in ZN staining, multiple species, apart from MTB, and even heads of sperm, exhibit acid-fast characteristics and can lead to inaccurate positive outcomes. Errors in the laboratory, such as the utilisation of scratched slides, aged stains, and contaminated water, can also generate inaccurate positive outcomes [17].

The results of our study indicate that the GeneXpert test, when conducted on EB samples of patients suspected of having FGTB, demonstrated a sensitivity of 100% (confidence interval (CI) 2.50–100.00%), a specificity of 99.5% (CI 97.23–99.99%), a PPV of 50% (CI 12.40–87.60%), and an NPV of 100% (CI 98.15–100.00%). The confidence interval for sensitivity (100%, CI 2.50–100.00%) was notably wide due to the small sample size. This was likely a consequence of the extremely low number of true positive cases (n=1) in the study. The small proportion of true positives means that even a slight change in case numbers can lead to considerable variations in sensitivity estimations. The result is less reliable due to the wide CI, which implies that the true sensitivity could be significantly lower than 100%. This suggests the need of a larger sample size to obtain a more precise estimate of sensitivity. Nonetheless, our findings of GeneXpert are in alignment with those of Kanti et al., where the sensitivity, specificity, PPV, and NPV were all 100% for GeneXpert for the detection of MTB in endometrial biopsy specimens, with histopathological examination as their reference [18]. In a study conducted by Sethi et al., it was found that the use of GeneXpert for MTB detection in endometrial aspirates had a specificity of 100% (CI 93.40–100%), with an NPV of 80.60% (69.11–89.24%). However, the sensitivity was found to be 0.00% (0.00–24.71%). The limited sensitivity of GeneXpert was attributed to a potential inefficient technique used in processing the material, which was ineffective in isolating bacterial DNA from the endometrial sample [14]. Consistent with our results, Mustafa et al. found that GeneXpert demonstrated a sensitivity of 84% and a specificity of 100% when utilised for the processing of EB specimens [19]. In addition, the Cohen’s Kappa statistic in our study indicated a substantial agreement between both the diagnostic methods, MGIT and GeneXpert, with a value of κ=0.66. The p-value of 0.047 (significant p-value <0.05) suggests that the observed agreement level (Kappa=0.66) is statistically different from zero. Essentially, the likelihood of the observed agreement being a result of random chance is minimal.

Ziehl-Neelsen staining, in our study showed a sensitivity of 0% (CI 0.00–97.50%), a specificity of 99% (CI 96.43–99.88%) and an NPV of 99.5% (99.49–99.50%). The results of our study correspond with those of Sethi et al., who also observed that ZN staining of the EB specimens had a sensitivity of 0% (CI 0.00–24.71%), a specificity of 100% (CI 93.40–100.00%), and an NPV of 80.60% (CI 69.11–89.24%). In order to obtain a positive ZN staining result, a concentration of at least 10^4^–10^6^ bacilli per ml of the specimen is necessary. However, due to FGTB being paucibacillary, it is highly improbable that ZN staining would yield an appropriate outcome [14]. In the present study, Cohen’s Kappa statistic indicated a lack of agreement between MGIT and ZN staining, with a κ value of –0.01. A p-value of 1, greater than 0.05, suggests that the observed level of agreement is not statistically significant. In simple terms, the probability is high that the agreement seen is a result of random chance. Hence, it is a poor method for the diagnosis of MTB, particularly in extrapulmonary specimens.

The precise prevalence of female genital tuberculosis (FGTB) is uncertain, despite numerous investigations, due to its asymptomatic nature and frequent inadvertent identification [13]. In our study, there was a 0.5% positivity rate for GTB, as validated by the gold standard method of MGIT culture, out of 200 women (ranging in age from 18 to 45 years) who agreed to participate in the study and fulfilled the inclusion criteria. The results of our study align with previous research conducted by Patel et al. in central India, who reported a prevalence rate of FGTB of 1.25% in women with infertility [13]. Similarly, Srinivas Rao et al. in Hyderabad identified a prevalence rate of 2.08%, Chatterjee et al. in Kolkata reported a rate of 1.6%, and Goel et al. in Delhi reported a rate of 3.7% [20], [21], [22]. However, in another study conducted by Naik et al., a 16% prevalence of probable genital tuberculosis was identified among women who reported infertility. The study implemented best clinical practices to diagnose GTB [23]. The low occurrence of positive cases of MTB in our study could be linked to the paucibacillary character of extra-pulmonary samples, unequal dispersion of tubercle bacilli in the material, a significant proportion of the bacilli in the specimen being in a bacteriologically inactive state, and the study being confined to our institution, hence restricting the overall significance of the results [24]. Nevertheless, FGTB should be a key consideration when assessing women with pelvic symptoms, including infertility, in high-TB-incidence regions like India or among individuals with epidemiological risk for TB (those who have resided in high-incidence areas, have a previous TB diagnosis, have been in contact with TB patients, or exhibit abnormal imaging indicative of TB) residing in low-incidence areas. Diagnostic delays in GTB may lead to considerable permanent organ damage and adverse reproductive results. Furthermore, assisted reproductive technologies may become the only method for conceiving a biological child, resulting in considerable costs. Therefore, an early diagnosis could avert such measures [23], [25].

There is no straight-forward diagnostic algorithm when it comes to the diagnosis of GTB. Multiple tests are frequently necessary to gather conclusive evidence for its diagnosis. It necessitates a strong suspicion based on clinical symptoms, radiological findings (such as hysterosalpingography and ultrasonography), and other investigations including the Mantoux test, erythrocyte sedimentation rate, tuberculosis foci on chest X-ray, and correlation with histopathological analysis, AFB in tissue section, and culture [13]. In addition, interferon gamma release assays (IGRAs) are blood tests designed to diagnose latent tuberculosis infection (LTBI) by measuring T-cell responses following overnight stimulation with antigens relatively distinctive to MTB. Utilising IGRAs as a primary screening instrument can enhance resource efficiency by minimising the necessity for more invasive examinations in low-risk situations. This method enables healthcare personnel to concentrate diagnostic efforts on patients most likely to benefit from further testing [26], [27]. A negative IGRA does not entirely rule out TB, but it could guide subsequent investigations such as endometrial biopsy or GeneXpert.

Our study is one of the few to use GeneXpert on endometrial samples to detect GTB. The innovative GeneXpert method uses automated nucleic acid amplification. It is a rapid, reliable, and cost-effective diagnostic method that can identify MTB and even rifampicin sensitivity, making it an extremely valuable tool for TB diagnosis. While its significance in the diagnosis of pulmonary tuberculosis has been recognised, its application to extrapulmonary tuberculosis is currently being studied [28]. Additionally, the NTEP provides free of cost TB diagnostic services at public health centres, including GeneXpert among a few others [29]. Our hospital, a tertiary care government facility, provides the same services to patients, the majority of whom are from low-income backgrounds. The low pricing in return helps sustain access in low-resource settings. However, private sectors may charge anything between 28.71 and 34.35 USD per sample, for GeneXpert testing [30]. 

The novel PCR-based Xpert MTB/CBNAAT has been employed in multiple studies to diagnose additional forms of extrapulmonary tuberculosis. Notwithstanding this, the utilisation of GeneXpert in the diagnosis of FGTB has been limited to a handful of studies, resulting in an extreme scarcity of data regarding its role in FGTB. GeneXpert is a valuable tool for promptly detecting FGTB. It has the additional benefits of rapid results, requiring only minimal technical competence, and the ability to identify drug-resistant TB strains. This is due to its high detection rate, as observed in our study as well. It exhibits greater sensitivity for both pulmonary and extrapulmonary TB compared to conventional smear microscopy and can be conducted on-site, with a turn-around time of only 2 hours. GeneXpert MTB/RIF, as a rapid point-of-care test, can swiftly identify rifampicin-resistant TB (RR-TB) compared to traditional drug-susceptibility testing, facilitating prompt intervention. It is an invaluable resource in the diagnosis of FGTB when used in conjunction with various other relevant findings and investigations, and ultimately result in better patient outcomes [31], [32], [33]. Most studies also emphasise on the integration of all diagnostic assays along with clinical correlation to diagnose FGTB, rather than relying on a single diagnostic test [13]. 

A significant limitation of our study was the relatively small sample size, which was a result of the limited time available for conducting the study. Although the size of our sample was small to offer confirmative evidence of GeneXpert’s effectiveness in detecting FGTB, it does offer data for further studies. Conducting additional research, specifically involving numerous centres with substantial sample sizes, on the use of GeneXpert as a diagnostic tool for female genital TB, will provide a more accurate assessment of its effectiveness and role. A comprehensive multicentric investigation of its efficacy may help identify rifampicin resistance early, reducing morbidity and mortality in susceptible individuals, especially in TB-endemic India.

## Conclusion

Our study has demonstrated only a small proportion (0.5%) of patients with FGTB in women presenting with primary and secondary infertility in the reproductive age groups at our institution. This could be owed to factors such as the paucibacillary nature of MTB in EPTB, specifically FGTB in our case, and the limited number of samples in the study.

Despite numerous studies, the exact prevalence of FGTB remains uncertain, primarily because of its asymptomatic nature and vague clinical presentations. The present study is among the few that has utilised GeneXpert MTB/RIF (CBNAAT) to aid in the diagnosis of FGTB. It has highlighted the promising results exhibited by GeneXpert in identifying *Mycobacterium* *tuberculosis* in FGTB with high sensitivity and specificity. Therefore, GeneXpert, being much faster and more feasible than conventional methods such as culture, could be incorporated into the standard evaluation of genital TB. It is a valuable addition to the current diagnostic tools for female genital TB, whose diagnostic algorithm is quite nuanced and ambiguous, especially in a TB-endemic country such as ours.

Lastly, there is a need for further research employing a large sample size, which could possibly and effectively demonstrate the practicality and potential of GeneXpert as a diagnostic modality for FGTB. 

## Notes

### Ethical statement

The study protocol followed the guidelines of the Declaration of Helsinki and was approved by Scientific Review Board & Ethics Committee of LHMC, New Delhi (letter no. SHKM/IEC/2019/161). Participants were enrolled after receiving written informed voluntary consent after explaining to them the nature of study in detail in their own language. Data confidentiality was maintained.

### Acknowledgments

The authors would like to thank all the patients who agreed to take part in the study and the technical staff of the Department of Microbiology, LHMC, New Delhi, India.

### Authors’ contributions

- Conceptualization: YR, NJ, MBJ
- Data curation: CKS, YR, NJ
- Formal analysis: YR, NJ, MBJ
- Investigation: CKS, YR, NJ
- Methodology: CKS, YR, NJ
- Project administration: CKS, YR, NJ
- Resources: CKS, YR, NJ, MBJ
- Software: CKS
- Supervision: YR, NJ, MBJ
- Validation: YR, NJ, MBJ
- Visualization: CKS, YR, NJ, MBJ
- Writing – original draft: CKS
- Writing – review & editing: CKS, YR, NJ, MBJ

### Competing interests

The authors declare that they have no competing interests.

## Figures and Tables

**Table 1:**
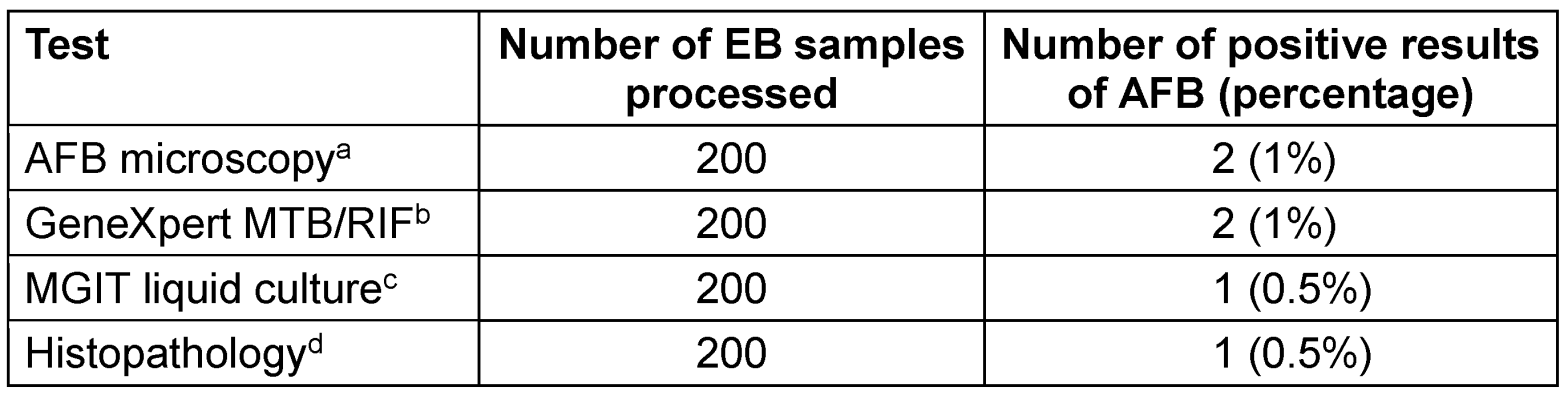
Results of various tests of endometrial biopsy (EB) samples for the detection of *Mycobacterium* *tuberculosis* (MTB) Legend: ^a^AFB microscopy utilising the Ziehl-Neelsen stain is a rapid and inexpensive technique to detect acid-fast bacilli (AFB), particularly MTB; ^b^Qualitative, nested real-time polymerase chain reaction (PCR) in vitro rapid diagnostic test for the detection of MTB; ^c^WHO-endorsed mycobacterial growth indicator tube (MGIT), a liquid culture using Middlebrook 7H9 broth that evaluates oxygen consumption by fluorescence for detection of MTB, gold-standard method for detection of AFB; ^d^Demonstration of epithelioid granuloma on histopathology, also gold-standard method [8].

**Table 2:**
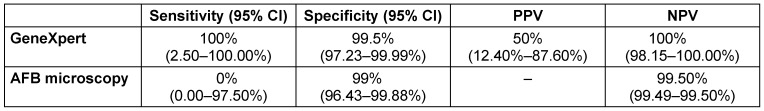
Sensitivity and Specificity, Positive Predictive Value (PPV) and Negative Predictive Value (NPV) of GeneXpert and AFB microscopy (ZN staining) for EB specimens in patients with infertility suspected of FGTB

**Table 3:**
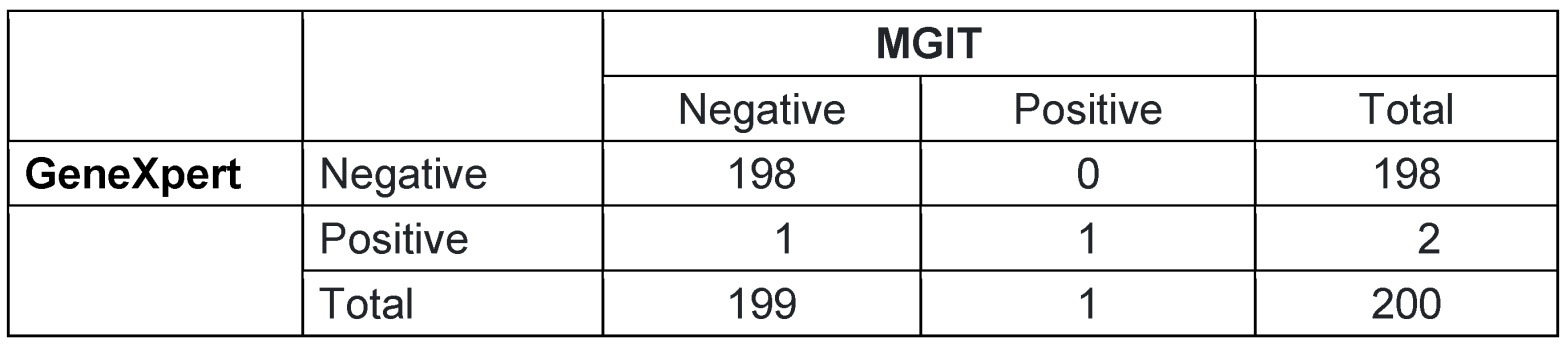
Agreement between GeneXpert MTB/RIF and MGIT liquid culture (n=200)

**Table 4:**
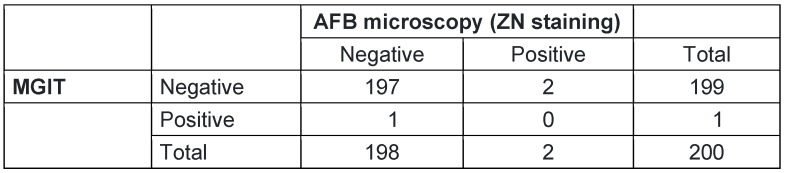
Agreement between AFB microscopy and MGIT liquid culture (n=200)
